# An electromagnetic field disrupts negative geotaxis in *Drosophila* via a CRY-dependent pathway

**DOI:** 10.1038/ncomms5391

**Published:** 2014-07-14

**Authors:** Giorgio Fedele, Edward W. Green, Ezio Rosato, Charalambos P. Kyriacou

**Affiliations:** 1 Department of Genetics, University of Leicester, Leicester LE1 7RH, UK

## Abstract

Many higher animals have evolved the ability to use the Earth’s magnetic field, particularly for orientation. *Drosophila melanogaster* also respond to electromagnetic fields (EMFs), although the reported effects are quite modest. Here we report that negative geotaxis in flies, scored as climbing, is disrupted by a static EMF, and this is mediated by cryptochrome (CRY), the blue-light circadian photoreceptor. CRYs may sense EMFs via formation of radical pairs of electrons requiring photoactivation of flavin adenine dinucleotide (FAD) bound near a triad of Trp residues, but mutation of the terminal Trp in the triad maintains EMF responsiveness in climbing. In contrast, deletion of the CRY C terminus disrupts EMF responses, indicating that it plays an important signalling role. CRY expression in a subset of clock neurons, or the photoreceptors, or the antennae, is sufficient to mediate negative geotaxis and EMF sensitivity. Climbing therefore provides a robust and reliable phenotype for studying EMF responses in *Drosophila*.

## Abstract (web summary)

The earth’s electromagnetic field has a modest effect on the behaviour of *Drosophila melanogaster*. Here, Fedele *et al*. use an assessment of climbing behaviour to describe how the blue-light circadian photoreceptor cryptochrome mediates a negative movement response to gravity in flies.

## Graphical abstract

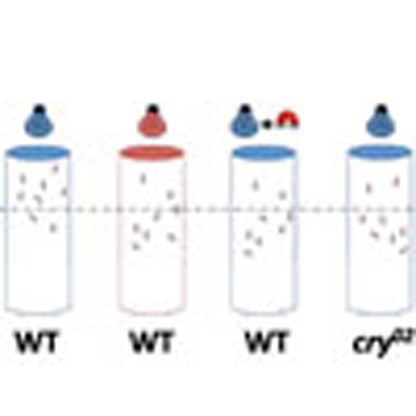

Many organisms have evolved the ability to sense and exploit the Earth’s magnetic field, particularly for navigation and orientation^1^. Three main models for magnetosensing have been promoted. Magnetic induction, which can only be applied to marine creatures, owing to the high conductivity of salt water^1^,^2^, the magnetite hypothesis that proposes a process mediated by crystals of permanently magnetic material (magnetite)^1^ and finally the radical pair mechanism (RPM), which relies on a chemical reaction involving specialized photoreceptors^3^,^4^.

In the RPM, the first step of the reaction requires absorption of a photon by the pigment molecule, leading to the transient formation of a radical pair of electrons in an overall singlet state (antiparallel spin orientation), in which the two unpaired electrons are at a suitable distance to undergo transition to the triplet state (parallel orientation). This transition may be sensitive to an electromagnetic field (EMF), altering the singlet-triplet balance. Return to the ground state can only occur from the singlet state, hence EMFs may alter the lifetime of the radical pair and any signal that it generates^3^,^4^. So far, the only photopigments proposed as putative candidates for the RPM are the cryptochromes (CRYs). These blue-light-sensing flavoproteins evolved from photolyases and are highly conserved across many different taxa^5^. CRYs are expressed in the eyes of mammals^6^ and migratory birds^7^, which are putative sites for magnetoreceptors in vertebrates^8^. In animals, CRYs also function as circadian photoreceptors in the *Drosophila* brain, mediating the light resetting of the 24 h clock^9^, but in vertebrates, the CRYs act as the main negative regulators for the circadian feedback loop^10^. The major difference between fly and vertebrate CRYs is that the former (type 1) are photosensitive, whereas the latter (type 2) are not^11^. Non-drosophilid insects can also encode CRY1 and CRY2’s, but CRY1s retain their light-sensing properties, whereas the CRY2s act as vertebrate-like negative regulators^12^.

Previous genetic analyses in *Drosophila* have suggested a CRY-dependent ability for magnetosensing^13^,^14^, whereas other fly studies have done so indirectly by utilizing wavelengths of light to which CRYs are sensitive^15^,^16^,^17^. The two experimental paradigms that utilized *cry* mutations in flies include a conditioning^13^,^18^ and a circadian behavioural assay^14^. In these studies, CRYs have been implicated as mediators of the fly’s EMF responses in a wavelength-dependent manner. Surprisingly, fly transformants carrying the *hCry2* transgene can also detect EMFs in the conditioning assay, suggesting that in the fly’s cellular environment, hCRY2 can be activated by light^19^. In addition, mutations of the terminal Trp residue, which forms the Trp triad believed to be important for mediating radical pair formation^20^, does not disrupt the EMF conditioning response, indicating that an unorthodox CRY-dependent EMF-sensing mechanism may be responsible^18^. Finally, although the CRYs implicate the circadian clock in magnetosensitivity, a working clock is not required for EMF responses in the fly conditioning assay^18^.

In the conditioning assay, the EMF behavioural effects are modest but consistent^13^,^18^,^19^, whereas the circadian period changes induced by EMF under blue constant light are highly variable, leading to shorter or longer periods in half the flies, and no response at all in the other half^14^. We therefore sought a different fly behavioural assay that might respond to EMFs with more marked and robust changes. Negative geotaxis in flies (their ability to climb against gravity) has been studied by both traditional quantitative genetic and modern genomic methods^21^. Artificial selection for flies that show high and low levels of geotaxis has been allied to transcriptomic analyses to reveal that CRY may play a significant role in this phenotype^21^, and CRY’s role in fly climbing behaviour has recently been confirmed^22^. We therefore suspected that this phenotype could be wavelength dependent and if so, might be compromised by applying an EMF. We show here that negative geotaxis is blue-light and CRY dependent and is significantly compromised by the application of a static EMF. We further reveal that the CRY C terminus is critical for mediating the effects of the EMF, and that CRY expression in specific clock neurons, eyes and antennae contribute to the EMF phenotype. We conclude that negative geotaxis provides a reliable method for studying behavioural responses to EMFs.

## Results

### Climbing is wavelength- and CRY-dependent

**Figure 1:**
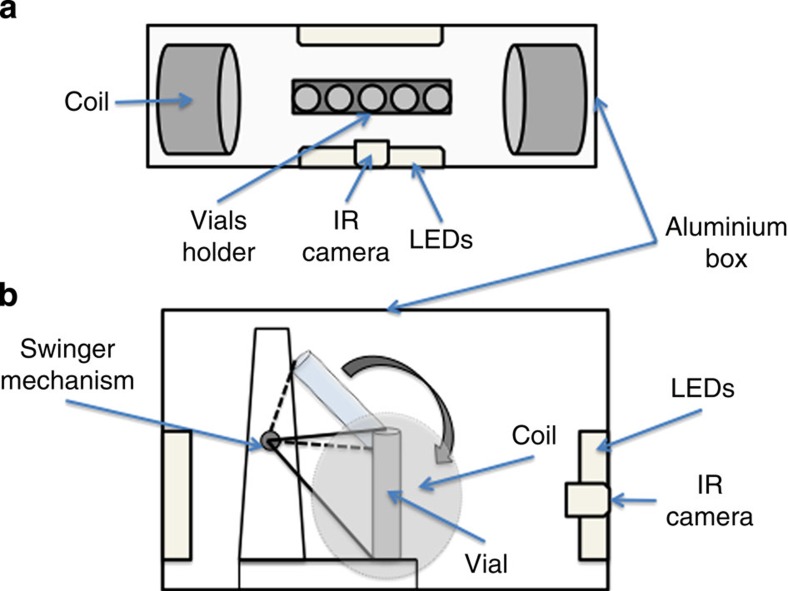
Measuring negative geotaxis under a static EMF. The delivery system for EMFs consists of a double-wrapped coil system (**a**, top view), and a custom-made swinger apparatus (**b**, side view) that allows tapping three vials simultaneously with equal force so the flies fall to the bottom of the tube. IR, infrared.

**Figure 2:**
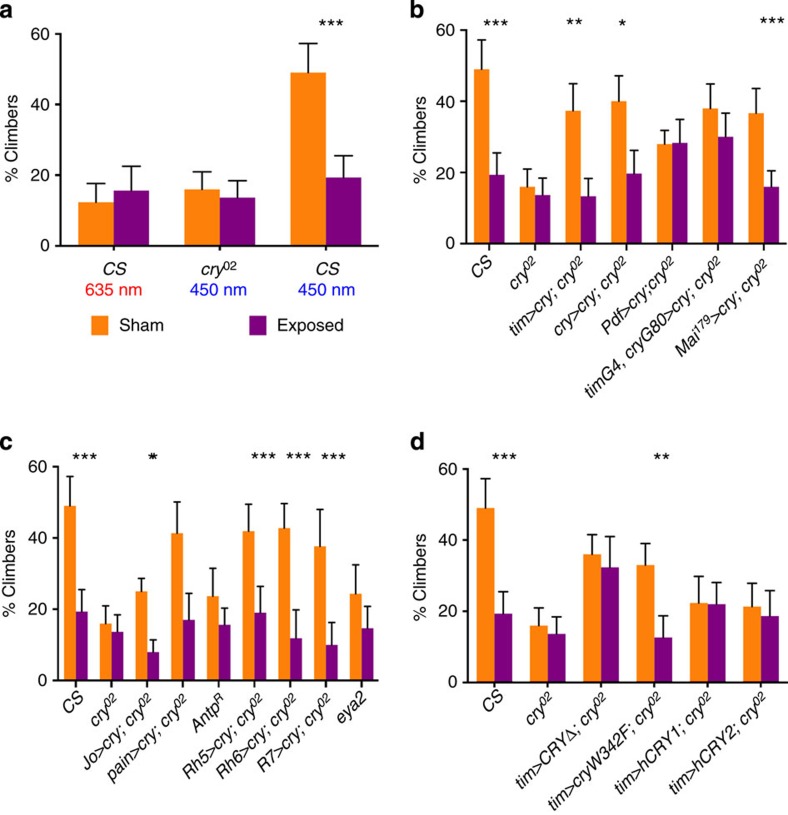
Negative geotaxis is CRY dependent and is sensitive to EMFs. Mean geotactic responses±s.e.m. based on three biological replicates. Orange bars, sham exposed; purple bars, EMF exposed. Asterisks denote results of Duncan’s *a posteriori* test within genotype after analysis of variance (ANOVA), *P<0.05, **P<0.01, ***P<0.001. The results from Canton-S (CS) and *cry*^*02*^ were used as positive and negative controls for all analyses and **b**–**d** represent experiments performed only at 450 nm. (**a**) Response of CS and *cry*^*02*^ exposed to different wavelengths of light. (ANOVA, genotype F_2,12_=16.48, *P*=0.00036, exposure F_1,12_=8.67, *P*=0.012, G × E interaction F_2,12_=9.86, *P*=0.002). *Post hoc* tests revealed significant differences only between CS in blue light under sham compared with all the other conditions (*P*<0.001). (**b**) Responses of clock *gal4/80>UAScry* genotypes on a *cry*^*02*^ background (ANOVA, genotype F_6,28_=3.98, *P*=0.005, exposure F_1,28_=36.1, *P*=2 × 10^−6^, G × E interaction F_6,28_=3.08, *P*=0.019. *Post hoc* tests reveal no significant differences between sham *timgal4/UAScry* or *crygal4/UAScry* compared with CS, nor for EMF exposure. For sham, *Pdfgal4>UAScry* vs *cry*^*02*^ *P*=0.1, vs CS *P*=0.009 *timgal4crygal80>UAScry* vs *cry*^*02*^ *P*=0.007, vs CS *P*=0.12; for EMF *Pdfgal4>UAScry* vs *cry*^*02*^ *P*=0.06, vs CS *P*=0.22, *timgal4crygal80>UAScry* vs *cry*^*02*^ *P*=0.039, vs CS *P*=0.16. (**c**) Responses of eye and antennal genotypes *(gal4>UAScry* on *cry*^*02*^ background) (ANOVA, genotype F_8,36_=5.45, *P*=0.00016, exposure F_1,36_=99.4, *P*~0, G × E interaction F_8,36_=3.25, *P*=0.007. *Post hoc* for sham, CS was not significantly different from sham *pain*, *rh5*, *rh6*, *R7gal4>UAScry*, but *JOgal4>UAScry* vs *cry*^*02*^ *P*=0.18, vs CS *P*=0.0005. For EMF, none of the genotypes were significantly different from CS or *cry*^*02*^). (**d**) Responses of *cry* variants driven by *timgal4* (ANOVA, genotype F_5,24_=6.89, *P*=0.0004, exposure F=_1,24_=16.8, *P*=0.0005 and G × E interaction F_5,24_=4.13, *P*=0.008. *Post hoc* sham *timgal4>cryΔ* vs *cry*^*02*^ *P*=0.007, vs CS *P*=0.04, *timgal4>cryW342F* vs *cry*^*02*^ *P*=0.02, vs CS *P*=0.017; for EMF *timgal4>cryΔ* vs *cry*^*02*^ *P*=0.01, vs CS *P*=0.06, *timgal4>cryW342F* vs *cry*^*02*^ *P*=0.2, vs CS *P*=0.33).

We examined climbing ability as the percentage of flies that could climb 15 cm in 15 s at different wavelengths using a custom-made apparatus (see Methods and Fig. 1). We used either a sham exposure or a static EMF of 500 μT, which although an order of magnitude greater than the Earth’s magnetic field, is an intensity comparable with that used in previous genetic studies of fly EMF sensitivity^13^,^14^. Figure 2a reveals that under blue light (450 nm), the proportion of wild-type Canton-S sham ‘climbers’ is significantly higher than in corresponding EMF exposed flies (*P*=0.0004), whereas in red light (635 nm) climbing is substantially reduced under sham exposure, to levels similar to those of EMF-exposed flies under blue light. We also investigated the *cry-null* mutant, *cry*^*02*^ in blue light, which reveals responses similar to wild-type flies in red light. We conclude that negative geotaxis requires both blue-light activation and the presence of CRY, and that climbing can be disrupted by a static EMF.

### Overexpressing CRY rescues EMF responses

**Figure 3:**
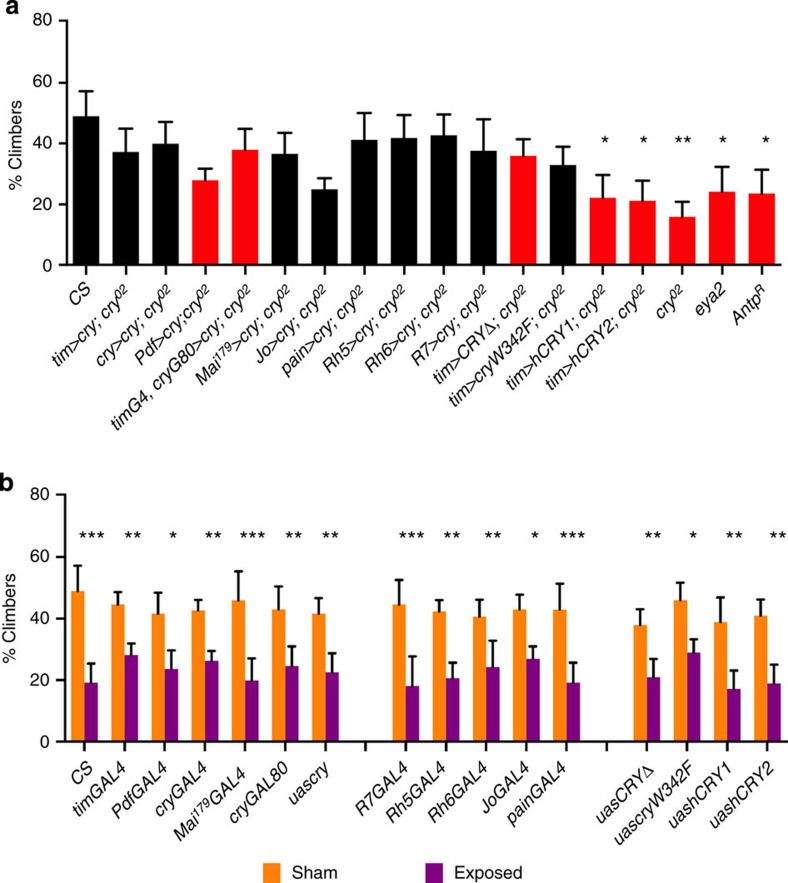
Control genotype responses to sham and EMFs. (**a**) Sham controls are shown to illustrate which genotypes did not respond (red bars) or did respond (black bars) to EMFs. Mean climbing scores (±s.e.m.) under blue light based on three biological replicates. Repeated measure analysis of variance (ANOVA) (F_(17,36)_=4.41, *P*=0.001) and Duncan’s *post hoc* analysis (*P<0.05, **P<0.01), further reveal which genotypes differ significantly in their sham responses to CS wild type. Note that *JO>cry; cry*^*02*^ have intermediate levels of climbing under sham, yet show an EMF response (Fig. 2c), whereas genotypes with higher levels of sham climbing (*Pdf>cry;cry*^*02*^, *timgal4*,*crygal80>cry;cry*^*02*^, *tim>cryΔ;cry*^*02*^) do not respond to EMF (Fig. 2b,d). (**b**) *GAL4/UAS* controls strains show normal EMF responses. Mean climbing scores (±s.e.m.) under blue light based on three biological replicates. Repeated measures ANOVA revealed a significant exposure (F_(1,64)_=217.52, *P*=0.0004) but no effect of genotype (F_(15,64)_=0.818, *P*=0.65) nor a G × E interaction (F_(15,64)_=0.60, *P*=0.86), so all genotypes responded in the same way to the EMF. All strains are in a *w*^*1118*^ genetic background. Duncan’s *post hoc* *P<0.05, **P<0.01, ***P<0.001.

We overexpressed fly CRY under GAL4 control, using various circadian clock drivers on a *cry*^*02*^ background. We observe that expressing CRY in most of the major clock neurons, using either the *timgal4* driver or in a more restricted *crygal4* pattern, restores high levels of climbing in sham, which was significantly reduced in EMF conditions, as in the wild type (Figs 2b and 3a). We also tested the climbing of all the gal4 driver and UAS lines that are used in this study, and all generate normal EMF responses (Fig. 3b). However, when CRY expression is restricted further using the *Pdfgal4* driver, which expresses in the lateral ventral (LNv) subset of clock neurons, intermediate levels of climbing are observed that are not further disrupted by EMF (Figs 2b and 3). A similar scenario prevails when the *timgal4crygal80* combination is used to drive CRY expression predominantly in the dorsal neurons plus three normally CRY-negative lateral dorsal neurons (LNds)^23^ with again, levels of climbing observed that are similar to those obtained with the *timgal4* and *crygal4* drivers, but with no significant reduction of geotaxis under EMF (Figs 2b and 3). In contrast to these restricted patterns of CRY expression, the *Mai*^*179*^*gal4* driver that expresses in the LNvs and three CRY-positive LNd neurons^23^,^24^ generated intermediate levels of climbing, which are nevertheless susceptible to an EMF. Consequently, it appears that among the canonical clock neurons, it is the three CRY-expressing LNd cells that are required to generate a robust EMF response.

We also investigated whether major peripheral tissues in the head, namely the eyes and antennae that normally express CRY, could also contribute to EMF sensitivity. The *rh5, rh6* and *R7gal4* eye-specific rhodopsin drivers all restore normal levels of climbing to *cry*^*02*^ mutants that are significantly reduced under EMF (Figs 2c and 3). To complement these results, the eyes-absent mutant, *eya*^*2*^, which has a complete absence of eyes, shows a significant reduction in climbing and no further reduction under EMF (Figs 2c and 3a). The antennal drivers *JOgal4* and *painlessgal4* also rescue the sham/EMF response on a *cry*^*02*^ background, in spite of the fact that in *JOgal4*, the sham level of climbing is significantly reduced compared with Canton-S flies (*P*=0.0005, Figs 2c and 3a) and no higher than that of *eya*^*2*^. Furthermore, the *Antp*^*R*^ mutant, in which antennae are transformed to mesothoracic legs, significantly reduces the climbing score under sham, but does not reduce it further under EMF (Figs 2c and 3a). These results suggest that the eyes and antennae also play significant roles in climbing and in the response to EMFs.

Finally, we expressed a number of *cry* variants on the *cry*^*02*^ background using *timgal4*, including human *hCry1* and *hCry2* transgenes, the latter having been reported to rescue the EMF effect on a conditioning paradigm^19^. Neither of these transgenes appears capable of rescuing the climbing phenotype beyond that of *cry*^*02*^, so they are not competent to respond to EMF (Fig. 2d). In contrast, a Trp to Phe mutation *(cryW342F)* of the terminal Trp of CRYs putative ‘Trp triad’, generates intermediate levels of climbing, which are significantly further reduced on EMF exposure (Figs 2d and 3). We also examined the sham/EMF response of the CRY C-terminal deletion mutant CRYΔ, which is constitutively active in both darkness and light^25^,^26^. Interestingly, under both sham and EMF conditions, this mutant shows intermediate levels of climbing but with no difference between the two conditions. Therefore, like *cryW342F*, *timgal4>cryΔ* retains the ability to climb but in contrast, is not responsive to an EMF, revealing a role for the CRY C terminus in magnetosensing (Figs 2d and 3a).

## Discussion

We have observed that *Drosophila* requires a functional CRY molecule to climb against gravity, confirming the results of two earlier studies that used different measures of negative geotaxis^21^,^22^. We have extended these observations by revealing that under blue light, the climbing of wild-type flies exposed to a 500-μT static EMF is significantly reduced compared with sham exposure. The pass/fail nature of our behavioural assay clearly differentiates between the two exposure conditions. In red light, flies exposed to sham or EMF show significantly reduced climbing, very similar to the levels observed under blue light with EMF exposure. Consequently, negative geotaxis is blue-light dependent, thereby implicating the fly’s dedicated circadian photoreceptor, CRY. As red light does not activate CRY, our results imply that EMFs compromise the photoreceptor’s response to blue light. Consistent with this, the *cry-null* mutant fails to climb in sham conditions, but this ability can be partially or almost fully rescued by overexpressing CRY in a number of different neuronal types that include clock neurons, antennae and eyes. These results suggest that CRY mediates the effects of EMFs, as also revealed in two other behavioural paradigms, a conditioning and a circadian assay^13^,^14^,^18^.

There is, nevertheless, a logical problem in the inference that CRY is the sensor for EMF taken only from the *cry-null* mutant data, in that the phenotype of climbing is itself CRY dependent, so any mutant that does not climb cannot show a reduction in climbing due to EMF. The same is true for the circadian EMF phenotype, where a change of period under constant dim blue light, which is CRY dependent, has been reported to be further modulated by EMF^14^. However, in *cry* mutants, as there is no initial circadian period change in blue light, there is no behavioural substrate for the EMF to modify. Thus *cry-null* mutants, in themselves, are not informative in these two assays. In contrast, in the conditioning assay, flies of various genetic backgrounds show both positive or negative naive preferences to an EMF, and this can be modulated by association with sucrose leading to an enhanced preference for EMF after training^13^,^18^. *cry-null* flies do not show any preference in the first place indicating they cannot sense the EMF, so they cannot be trained, thus there is no net change in preference after training. Yet a strong indication for the role of *cry* in this phenotype is provided by the *cry-null* mutant’s initial inability to sense the EMF, which is independent of the type of *cry-null* allele and the flies’ genetic background^13^,^18^.

With this reservation in mind, perhaps the most convincing support for the CRY-EMF hypothesis in our climbing assay requires a mutant that climbs in sham conditions to near wild-type levels, so that the CRY molecule retains basic geotactic function, yet would climb to similar levels under EMF, reflecting a mutant, suppressed EMF response. One mutation that fulfils this requirement is CRYΔ, which although producing an unstable CRY, retains some residual molecular response to light^25^,^26^. This mutant shows intermediate levels of climbing between *cry*^*02*^ and wild type under sham conditions, but does not respond to the EMF by reducing its climbing. The CRY C-terminal region may therefore play a pivotal role in the intracellular signalling of the CRY response to EMF, possibly by modulating downstream protein–protein interactions.

Another mutation *cryW342F* that substitutes a Phe for the terminal Trp in the putative ‘Trp triad’ that is a candidate for mediating radical pair formation, shows similar levels of climbing to *cryΔ*, but in sharp contrast, is responsive to EMF. This result echoes the observation that *cryW342F* is also able to retain EMF sensitivity in the conditioning assay^18^. Consequently, the terminal CRY Trp342 may not be the critical residue that is a prerequisite for the RPM, and perhaps another residue within that local conformation is involved, perhaps a tyrosine^27^. While both the climbing and conditioning assays reveal consistent effects for the terminal Trp mutant, the same could not be stated for hCRY2. In the conditioning test, hCRY2 is EMF-sensitive^19^, but in the climbing assay, hCRY1 and hCRY2 behave very similarly to *cry*^*02*^, suggesting that they are not blue-light responsive, revealing that the ability of *hCRY* to rescue an EMF response is phenotype dependent.

We also obtained EMF phenotypes when we varied the expression patterns of CRY. Under the control of different clock drivers, we observed that as we reduced expression from *timgal4* (expressed in nearly all clock cells) to *crygal4* (only CRY-expressing cells), to *Pdfgal4* (expressed in LNvs) and *timgal4;crygal80* (predominantly dorsal neurons, DNs and three LNds that do not normally express high levels of CRY), we noticed that under sham conditions the proportion of climbers was generally either intermediate between the mutant and wild-type values or not statistically different from the value of the wild type. For example, *timgal4;crygal80* gave 38% climbers compared with *cry*^*02*^ 16% and wild type, 49%. Yet for the *Pdfgal4* and *timgal4;crygal80* drivers there were no significant differences between the sham and EMF conditions, so the EMF response had been lost. However, the *Mai*^*179*^*gal4* driver, which expresses in the LNvs and three strongly CRY-positive LNd cells^23^,^24^, restored the intermediate levels of climbing under sham control as well as the EMF suppression. Comparing this result with that of the *timgal4crygal80* combination and *Pdfgal4* drivers, it would appear that CRY expressed in the three CRY-positive LNd neurons could be sufficient for restoring both climbing and the EMF responses. The LNd cluster is involved in circadian locomotor responses under light conditions^28^, providing a rationale for why they may play an important role in the climbing phenotype under blue light. Our results thus provide a new, non-circadian function for the CRY-positive LNds.

The clock neurons are not the only relevant cells for mediating the effects of EMFs. CRY expression in the R8 photoreceptors of pale ommatidia (via *rh5gal4*), or in the R8 yellow ommatidia^29^ and the Hofbauer-Buchner eyelet (*rh6gal4*)^30^ or in the R7 cell, is sufficient for robust climbing and EMF responses. Johnston’s organ (JO), which is located in the second antennal segment, has been previously implicated in negative geotaxis^31^ and our results with *JOgal4*, which expresses specifically in JO^31^,^32^, and *paingal4*, which is more widely expressed in the antennae and some central neurons^33^, suggest that CRY expression in JO is sufficient for mediating the effects of EMF. Consequently, there are three separate anatomical foci (LNds, eyes and antennae), where CRY expression in any one is sufficient to restore EMF sensitivity to *cry* mutants. While this might suggest some type of cellular redundancy, severe mutations of the eyes or the antenna, which reduce the climbing response to ~30% do not give a significant further reduction in geotaxis when exposed to EMF. While this might reflect the general behavioural effects of neurological damage in structures that might be required to be intact (even if CRY negative) to generate normal geotactic responses, a similar level of sham climbing is observed in *JO>cry; cry02* flies, which are nevertheless significantly disrupted in climbing on EMF exposure (Fig. 2c). Thus an integrative scenario is suggested, where in the anatomical absence of one structure, CRY expression in the other two cannot compensate to generate an EMF response.

In other insects such as the Monarch butterfly, the antennae play a prominent role in orientation and migration^34^,^35^,^36^, but it remains to be seen whether *Drosophila*’s ability to respond to magnetic fields has any adaptive function. One well-known switch in geotactic behaviour occurs in the late larval stage, whereby larvae that have spent most of their development digging down into food (positive geotaxis) become negatively geotactic in the late 3rd larval instar before they pupate. The adult’s escape response also involves negative geotaxis, yet whether the Earth’s magnetic field (or CRY) plays any role in these adaptive phenotypes has not been studied, to our knowledge. In conclusion, our results have identified a novel and robust CRY-dependent behavioural phenotype in *Drosophila* that responds to EMFs, and which may be extremely useful for further neurogenetic dissection of the cellular and molecular basis of magnetosensitivity.

## Methods

### Fly strains

Flies were maintained in LD 12:12 at 25 °C. Canton-S flies, *cry*^*02*^ and all *gal4* drivers and mutants (including *UAS* transgenes) were backcrossed into a *w*^*1118*^ background for 5–7 generations. *timGAL4*, *UAScry24b* and *UASHAcry* and CRYΔ mutants (refs 26, 37) were further crossed into a *cry*^*02*^ background^38^ using standard balancing techniques. *JOgal4* and *crygal80* were recombined onto the third chromosome carrying *cry*^*02*^. *UASmychCRY1/2* and *cryW342F* strains were obtained from Steven Reppert (University of Massachusetts). *R7gal4*, *rh5gal4*, *rh6gal4*, *R7gal4*, *Antp*^*R*^, *paingal4 and eya*^*2*^ strains were obtained from the Bloomington stock centre (IN, USA). *Mai*^*179*^*gal4* was obtained from Francois Rouyer (Gif, Paris).

### Behavioural apparatus

An EMF delivery system was designed consisting of an aluminium box placed in a temperature-controlled room, containing two double-wrapped (50 windings each) Helmholtz coils^39^ that allow sham and EMF exposures to be generated. A constant static magnetic field of 500 μT was produced by the coils through a power pack (Fig. 1a). Ten, 2–3-day-old males were placed in a plastic vial and tapped to the bottom by means of a custom-made ‘swinger’ that allowed three vials to be tapped to the bottom simultaneously with exactly equal force (Fig. 1b). An infrared webcam (Logitech) was used to film the flies. Flies that were able to reach a vertical height of 15 cm in 15 s were counted as ‘climbers’, and each tube was tested 10 times, with 30 s between each of the first 5 trials, then after a 15-min rest, another 5 trials were performed. The EMF or sham was applied at random after every group of 10 trials. Each set of 10 trials on the swinger ran three different genotypes simultaneously in the three tubes. Experiments were run at 25 °C either in dim blue (450 nm, 40 nm range) or dim red light (635 nm, 20 nm range) using light-emitting diodes with an intensity at the surface of the vials of 0.25 μW cm^−2^. Three biological replicates were used for each genotype, and data were analysed using a multifactorial analysis of variance with repeated measures^40^. All statistical analyses in this study were performed using GraphPad Prism version 6.00 for Windows, (GraphPad Software, La Jolla, CA, USA) and STATISTICA (data analysis software system, version 8.0, StatSoft Inc. 2008).

## Author contributions

G.F. performed the experiments; E.W.G. and G.F. designed the apparatus; E.R. and C.P.K. supervised the work; C.P.K. and G.F. analysed the data and co-wrote the manuscript.

## Additional information

**How to cite this article**: Fedele, G. *et al*. An electromagnetic field disrupts negative geotaxis in *Drosophila* via a CRY-dependent pathway. Nat. Commun. 5:4391 doi: 10.1038/5391 (2014).
